# Bridging the Gap in Emergency Medicine in Pakistan

**DOI:** 10.12669/pjms.36.ICON-Suppl.1726

**Published:** 2020-01

**Authors:** Syed Ghazanfar Saleem, Kaniz Farwa Haider, Zayed Yasin, Megan Rybarczyk

**Affiliations:** 1 Consultant, Department of Emergency Medicine, The Indus Hospital, Karachi, Pakistan; 2 Program Associate, Certification Program in Emergency Medicine, Global Health Directorate, Indus Health Network, Karachi, Pakistan; 3 Consultant Emergency Physician, CPS Medical Group; Faculty Lead, Certificate Program in Emergency Medicine, Global Health Directorate, Indus Health Network, Karachi, Pakistan; 4 Department of Emergency Medicine, Brigham and Women’s Hospital, Harvard Medical School, Boston, USA

Emergency conditions like acute cardiovascular disease, trauma, and stroke dominate the 10 leading causes of mortality in Pakistan.^1^ The Emergency Medicine (EM) specialist density in Pakistan is 0.02 physicians per 100,000 people, with 11 and 3 physicians per 100,000 in the US^2^ and UK^3^ respectively. Pakistan’s dearth of structured EM training programs has caused a persistent gap in quality emergency care provision, exacerbated by a high turnover rate. A dedicated EM workforce will take generations to develop, hence training non-specialist Emergency Department (ED) physicians should be prioritized in the interim.

A one-year Certification Program in Emergency Medicine (CPEM) was thus developed by specialists from The Indus Hospital (TIH), Karachi and Brigham and Women’s Hospital – a teaching affiliate of Harvard Medical School, USA – and launched at TIH in July 2018. CPEM aims to train ED physicians in proper emergency care, improve ED employee retention, and kindle support for EM nationally. CPEM’s curriculum is derived from national and international EM guidelines and expert feedback.

CPEM offers didactic and practical learning, supervised by local leadership and international faculty rotated monthly. Visiting faculty in the first year included consultants from the USA, UK, Australia, Saudi Arabia and Malawi. Participants are assessed through regular examinations, and formative and summative evaluations. Educational innovations include practical workshops (ultrasound, procedures), flipped-classroom learning, weekly case-based discussions over a messaging application, and use of low-cost simulation models to hone procedural skills.

In June 2019, CPEM graduated 27 participants (out of 32 originally enrolled) who are expected to understand fundamental EM concepts and ED processes, seek formal specialisation, represent and advocate for EM over various platforms, and provide improved patient care. It is hoped that the expansion of CPEM’s model to other hospitals, the inter-ED collaboration, and continued interest in EM will help to significantly advance emergency care’s quality and accessibility in Pakistan.
